# Machine learning prediction of metabolic dysfunction-associated fatty liver disease risk in American adults using body composition: explainable analysis based on SHapley Additive exPlanations

**DOI:** 10.3389/fnut.2025.1616229

**Published:** 2025-06-30

**Authors:** Yan Hong, Xinrong Chen, Ling Wang, Fan Zhang, ZiYing Zeng, Weining Xie

**Affiliations:** ^1^Affiliated Guangdong Hospital of Integrated Traditional Chinese and Western Medicine of Guangzhou University of Chinese Medicine, Guangzhou University of Chinese Medicine, Foshan, China; ^2^First Clinical Medical College, Guangzhou University of Chinese Medicine, Guangzhou, China; ^3^First Affiliated Hospital of Guangxi University of Chinese Medicine, Nanning, China; ^4^Infectious Disease Department, Guangdong Provincial Hospital of Integrated Traditional Chinese and Western Medicine, Foshan, China

**Keywords:** MAFLD, body composition, machine learning, SHAP, NHANES

## Abstract

**Background:**

Metabolic dysfunction-associated fatty liver disease (MAFLD) is a prevalent and progressive liver disorder closely linked to obesity and metabolic dysregulation. Traditional anthropometric measures such as body mass index (BMI) are limited in their ability to capture fat distribution and associated risk. This study aimed to develop and validate machine learning (ML) models for predicting MAFLD using detailed body composition metrics and to explore the relative contributions of adipose tissue features through explainable ML techniques.

**Methods:**

Data from the 2017–2018 National Health and Nutrition Examination Survey (NHANES) were used to construct predictive models based on anthropometric, demographic, lifestyle, and clinical variables. Six ML algorithms were implemented: decision tree (DT), support vector machine (SVM), generalized linear model (GLM), gradient boosting machine (GBM), random forest (RF), and XGBoost. The Boruta algorithm was used for feature selection, and model performance was evaluated using cross-validation and a validation set. SHapley Additive exPlanations (SHAP) were employed to interpret feature contributions.

**Results:**

Among the six models, the GBM algorithm exhibited the best performance, achieving area under the receiver operating characteristic curve (AUC) values of 0.875 (training) and 0.879 (validation), with minimal fluctuations in sensitivity and specificity. SHAP analysis identified visceral adipose tissue (VAT), BMI, and subcutaneous adipose tissue (SAT) as the most influential predictors. VAT had the highest SHAP value, underscoring its central role in MAFLD pathogenesis.

**Conclusion:**

This study demonstrates the effectiveness of integrating body composition features with machine learning techniques for MAFLD risk prediction. The GBM model offers robust predictive accuracy and interpretability, with potential applications in clinical decision-making and public health screening strategies. SHAP analysis provides meaningful insights into the relative importance of adiposity measures, reinforcing the value of fat distribution metrics beyond conventional obesity indices.

## Introduction

Nonalcoholic fatty liver disease (NAFLD) is a chronic and progressive liver disorder that develops in genetically susceptible individuals in the context of nutritional excess and insulin resistance (IR). The disease spectrum ranges from simple steatosis (nonalcoholic fatty liver, NAFL) to nonalcoholic steatohepatitis (NASH), and may progress to advanced stages such as fibrosis and cirrhosis (1). With the discovery of a strong relationship between NAFLD and metabolic risk factors, it has been renamed in recent years as metabolic dysfunction-associated fatty liver disease (MAFLD) and metabolism-associated steatosis liver disease (MASLD). MASLD is defined as hepatic steatosis accompanied by cardiometabolic abnormalities, in the absence of other causes of steatosis or excessive alcohol consumption (≥30 g/day for men and ≥20 g/day for women) (2). By contrast, the 2020 diagnostic criteria for MAFLD (3–6), focus more on metabolic abnormalities than alcohol intake (7). Recent meta-analyses have estimated the global prevalence of MAFLD to be as high as 38.77%, which significantly exceeds the prevalence reported under the previous NAFLD criteria (8–10).

MAFLD is strongly associated with an increased risk of atherosclerotic cardiovascular disease (CVD), chronic kidney disease (CKD), hepatic decompensation, and hepatocellular carcinoma (HCC) (11, 12). Emerging evidence suggests that the “liver–spleen axis” plays a critical role in the pathogenesis and progression of MAFLD. Splenomegaly has been positively correlated with central obesity and the severity of hepatic steatosis (13, 14). Animal studies have shown that high-fat diets induce splenic sinusoidal dilation and lipid accumulation in mice, whereas splenectomy significantly increases hepatic immune cell infiltration and the expression of proinflammatory cytokines such as IL-6 and TNF-*α* (15, 16). These findings suggest that the spleen may play a protective role in metabolic regulation by maintaining immune homeostasis and attenuating excessive inflammatory responses. Moreover, MAFLD is strongly associated with obesity-related chronic inflammation (17). Dysfunctional adipose tissue promotes the release of free fatty acids (FFAs), which exacerbate hepatic steatosis by inducing inflammation and promoting the development of IR (18).

In clinical practice, BMI is widely used to assess general obesity due to its simplicity (19, 20). However, BMI cannot differentiate between fat mass and lean mass, nor does it account for the spatial distribution of adipose tissue (21, 22). Studies have demonstrated that obesity-related metabolic disturbances are closely associated with fat distribution patterns, particularly the accumulation of visceral adipose tissue (VAT) (23–26). Total abdominal fat area (TAFA) has been identified as an independent risk factor, exhibiting stronger associations with CVD, metabolic disorders, and all-cause mortality than BMI (26–29). TAFA is composed of both subcutaneous adipose tissue (SAT) and VAT. SAT can expand physiologically to buffer against ectopic lipid deposition; however, its compensatory capacity may be constrained by genetic predisposition or impaired adipogenesis. Persistent caloric excess results in pathological accumulation of VAT (30, 31). VAT is regarded as a hallmark of metabolically unhealthy obesity and is independently associated with a wide range of metabolic disturbances (32–34). Its abnormal expansion is indicative of ectopic lipid deposition (35, 36). The visceral-to-subcutaneous fat ratio (VSR), a novel adiposity metric, has been strongly associated with elevated levels of proinflammatory cytokines and the progression of hepatic steatosis (37).

As a subfield of artificial intelligence, machine learning (ML) excels at identifying complex nonlinear relationships within high-dimensional datasets and has shown considerable advantages in disease screening and risk assessment (38, 39). Unlike traditional statistical methods, ML does not require assumptions about variable distributions and is well-suited to capturing intricate interactions and nonlinear associations. Although ML has been increasingly applied in the diagnosis of liver diseases (22, 40, 41), its utility in exploring associations between multidimensional obesity indices and MAFLD remains underinvestigated. In this study, we leveraged data from the National Health and Nutrition Examination Survey (NHANES) to identify obesity-related indices strongly associated with MAFLD using ML techniques. Furthermore, we employed SHapley Additive exPlanations (SHAP) to interpret the contribution of individual features and to develop an interpretable predictive model.

## Methods

### Participants

**Figure 1:**
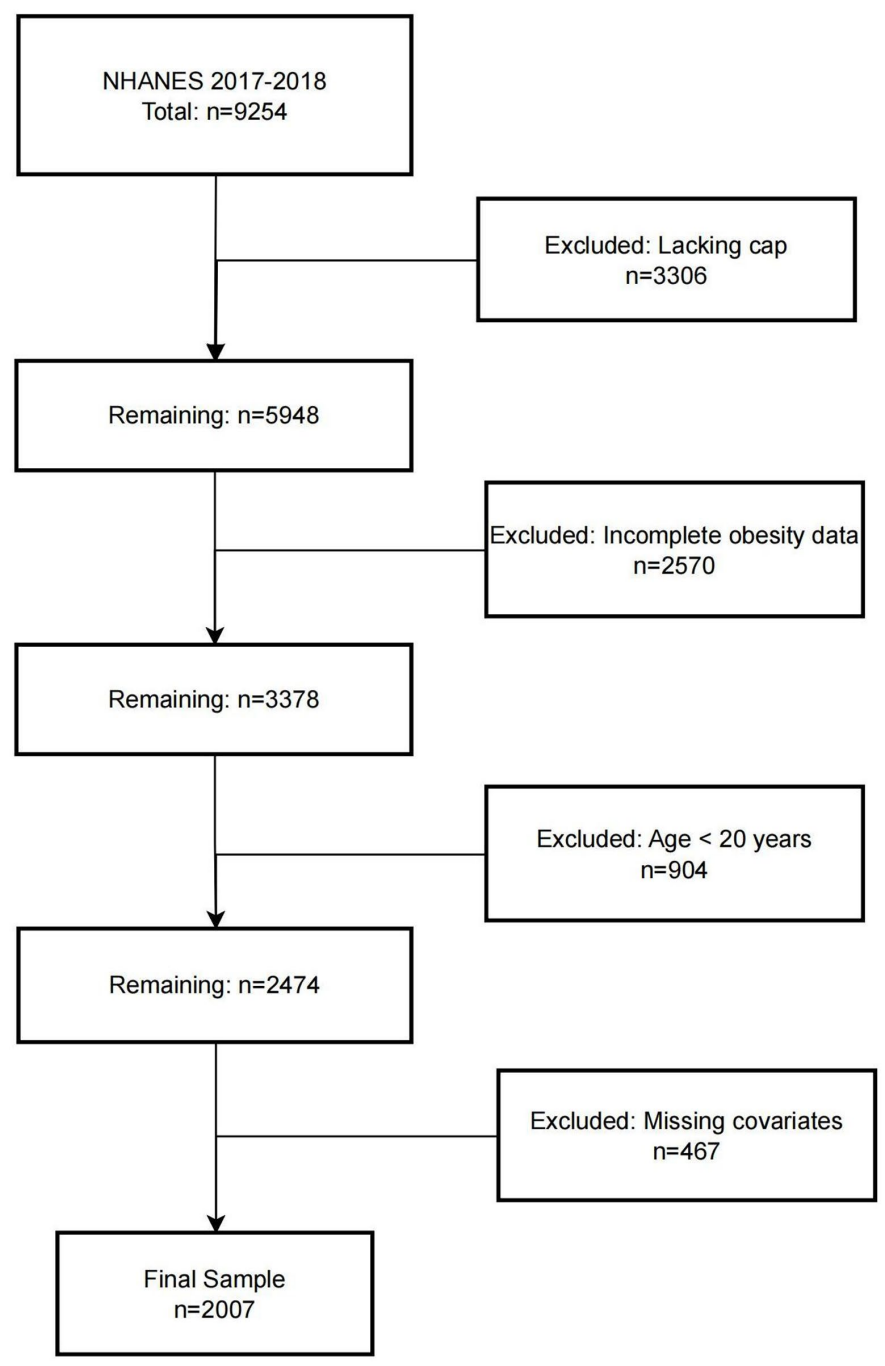
Flowchart.

The National Health and Nutrition Examination Survey (NHANES) is a nationally representative program jointly conducted by the Centers for Disease Control and Prevention (CDC) and the National Center for Health Statistics (NCHS). The study protocol was approved by the NCHS Research Ethics Review Board, and written informed consent was obtained from all participants. In this study, data from the 2017–2018 NHANES cycle were analyzed. The initial cohort consisted of 9,254 participants. Individuals were sequentially excluded based on the following criteria: (1) lack of hepatic steatosis assessment (*n* = 3,306); (2) missing obesity-related measurements (*n* = 2,570); (3) age < 20 years (*n* = 904); and (4) incomplete covariate data (*n* = 467). A total of 2,007 participants were ultimately included in the final analysis. The screening and selection process is presented in Figure 1.

### Definition of MAFLD

MAFLD was diagnosed according to the international expert consensus criteria established in 2020 (7). Hepatic steatosis was assessed using the controlled attenuation parameter (CAP) measured by the FibroScan® 502 V2 Touch device, with a CAP value ≥274 dB/m considered indicative of hepatic steatosis (42). In addition, diagnosis required the presence of at least one of the following three conditions:

1. Overweight or obesity: defined as a BMI ≥ 25 kg/m^2^ for Caucasian individuals or ≥23 kg/m^2^ for Asian individuals.
2. Type 2 diabetes mellitus (T2DM): diagnosed based on any of the following criteria: (a) fasting plasma glucose (FPG) ≥ 7.0 mmol/L; (b) glycated hemoglobin (HbA1c) ≥ 6.5%; (c) a clinical diagnosis of diabetes by a qualified physician.
3. Metabolic dysregulation: defined as the presence of at least two of the following seven criteria:
  (1) Waist circumference ≥102 cm in men or ≥88 cm in women (or ≥90 cm in Asian men or ≥80 cm in Asian women).
  (2) Blood pressure ≥130/85 mmHg or current use of antihypertensive medication.
  (3) Plasma triglycerides ≥150 mg/dL or treatment with lipid-lowering agents.
  (4) Plasma high-density lipoprotein (HDL) cholesterol <40 mg/dL in men or <50 mg/dL in women, or use of lipid-modifying therapy.
  (5) Prediabetes (FPG 5.6–6.9 mmol/L; 2-h post-load glucose 7.8–11.0 mmol/L; or HbA1c 5.7–6.4%).
  (6) Homeostasis model assessment of insulin resistance (HOMA-IR) ≥ 2.5.
  (7) High-sensitivity C-reactive protein (hs-CRP) > 2 mg/L (7).

### Definition of body composition

Anthropometric measurements were conducted by trained NHANES personnel at Mobile Examination Centers (MEC) following standardized protocols. TAFA(g), VAT(g), and SAT(g) were measured using dual-energy X-ray absorptiometry (DXA), with data automatically processed by Hologic APEX software. The VSR was calculated as the ratio of VAT to SAT. BMI was assessed by trained staff using calibrated instruments to measure height and weight, and was calculated using the formula: weight (kg) / height^2^ (m^2^).

### Covariates

Data extracted from the NHANES database included the following covariates:(1) Demographic characteristics: age, gender (male or female), educational attainment (less than 9th grade; 9th–11th grade; high school graduate or GED equivalent; some college or associate degree; college graduate or above), race(Mexican American; other Hispanic; non-Hispanic White; non-Hispanic Black; other race, including multiracial), marital status (married; widowed; divorced; separated; never married; living with a partner), and family income–poverty ratio (PIR). (2) Lifestyle factors: Alcohol consumption was defined as the intake of more than two standard drinks per day over the past 12 months. Smoking status was defined as having smoked at least 100 cigarettes in a lifetime. (3) Laboratory and clinical measures: These included HbA1c, FPG, HDL, and low-density lipoprotein (LDL). Hypertension was defined as having a systolic blood pressure ≥140 mmHg and/or diastolic blood pressure ≥90 mmHg based on three separate readings, or a clinical diagnosis of hypertension. Diabetes mellitus was defined as HbA1c > 6.5% or FPG > 7.0 mmol/L.

### Statistical analysis

All statistical analyses were performed using R software (version 4.3.2) and EmpowerStats. Baseline characteristics were summarized according to MAFLD status. Continuous variables were expressed as mean ± standard deviation (SD), while categorical variables were presented as counts with corresponding percentages. Data visualization was conducted using the ggplot2 package, generating bar plots for categorical variables and histograms for continuous variables. A Pearson correlation matrix was constructed to illustrate inter-variable correlations. To assess multicollinearity, the variance inflation factor (VIF) was calculated through iterative regression modeling. Variables with VIF values exceeding 10 were excluded from subsequent analyses. Feature selection was conducted using the Boruta algorithm, which utilizes shadow features based on random forests to identify the most relevant predictors. A *Z*-score boxplot was used to visualize feature importance, and the top 10 features most strongly associated with MAFLD were retained for modeling. Prior to model construction, the dataset was randomly partitioned into a training set (70%) and a validation set (30%). Six ML models were developed using the caret package: decision tree (DT), support vector machine (SVM), generalized linear model (GLM), gradient boosting machine (GBM), random forest (RF), and eXtreme Gradient Boosting (XGBoost). All models were trained using 10-fold cross-validation on the training dataset. Model performance was evaluated based on the following metrics: area under the receiver operating characteristic curve (AUC), accuracy, sensitivity, specificity, F-beta score, and area under the precision-recall curve (AUPRC) metrics. For comparisons between models, ANOVA was applied to normally distributed performance data, while the Kruskal–Wallis test was used for non-normally distributed variables. To further validate model generalizability, retraining was conducted on the validation set. Model interpretability and performance were assessed using the DALEX package, which generated explanatory plots and diagnostic measures. Receiver operating characteristic (ROC) curves were constructed to assess discriminatory ability. In addition, residual boxplots were plotted to visualize residual distributions, while PR curves were employed to evaluate the trade-off between precision and recall across the models.

Finally, the best-performing model was selected based on the AUC as the primary evaluation metric, supplemented by additional performance indicators. To enhance model interpretability, SHAP analysis was subsequently employed to quantify the contribution of each feature within the optimal model.

## Results

### Baseline characteristics

A total of 2,007 participants were included in the final analysis, of whom 1,004 were diagnosed with MAFLD. Compared to participants without MAFLD, those with MAFLD were significantly older, had a higher proportion of males, and were more likely to be of Mexican American or non-Hispanic White ethnicity. In terms of marital status, the majority of MAFLD participants were married. With respect to obesity-related indices, the MAFLD group exhibited significantly higher levels of BMI, SAT, VAT, VSR, and TAFA. Furthermore, the prevalence of hypertension and diabetes mellitus was substantially higher among participants with MAFLD compared to those without the condition (Table 1).

**Table 1:** Baseline population table.

Variable	Non MAFLD	MAFLD	*p*-value
*N*	1,003	1,004	
Age (years)	37.368 ± 11.557	42.744 ± 11.034	<0.001
Gender (%)			0.017
Male	479 (47.757%)	533 (53.088%)	
Female	524 (52.243%)	471 (46.912%)	
Race (%)			0.002
Mexican American	112 (11.167%)	171 (17.032%)	
Other Hispanic	87 (8.674%)	80 (7.968%)	
Non-Hispanic White	319 (31.805%)	328 (32.669%)	
Non-Hispanic Black	233 (23.230%)	195 (19.422%)	
Other Race -Including Multi-Racial	252 (25.125%)	230 (22.908%)	
Education (%)			0.021
Less than 9th grade	39 (3.888%)	57 (5.677%)	
9–11th grade	112 (11.167%)	105 (10.458%)	
High school graduate/GED or equivalent	231 (23.031%)	222 (22.112%)	
Some college or AA degree	329 (32.802%)	377 (37.550%)	
College graduate or above	292 (29.113%)	243 (24.203%)	
Marital status (%)			<0.001
Married	446 (44.467%)	534 (53.187%)	
Widowed	12 (1.196%)	18 (1.793%)	
Divorced	73 (7.278%)	100 (9.960%)	
Separated	28 (2.792%)	46 (4.582%)	
Never married	312 (31.107%)	194 (19.323%)	
Living with partner	132 (13.161%)	112 (11.155%)	
PIR	2.586 ± 1.658	2.595 ± 1.636	0.774
BMI(kg/m^2^)	25.335 ± 4.891	32.546 ± 6.508	<0.001
SAT(g)	1237.871 ± 657.954	2032.317 ± 783.027	<0.001
TAFA(g)	1573.288 ± 773.010	2671.213 ± 887.613	<0.001
VAT(g)	335.418 ± 184.246	638.896 ± 249.973	<0.001
VSR	0.324 ± 0.196	0.348 ± 0.157	<0.001
Hypertension (%)			<0.001
Non Hypertension	846 (84.347%)	581 (57.869%)	
Hypertension	157 (15.653%)	423 (42.131%)	
Diabetes (%)			<0.001
Non diabetes	959 (95.613%)	827 (82.371%)	
Diabetes	44 (4.387%)	177 (17.629%)	
Smoking status (%)			0.240
Non-smoking	631 (62.911%)	606 (60.359%)	
Smoking	372 (37.089%)	398 (39.641%)	
Alcohol drinking (%)			0.933
Non-drinking	710 (70.788%)	709 (70.618%)	
Drinking	293 (29.212%)	295 (29.382%)	

PIR, Household Income-Poverty Ratio; BMI, Body mass index; VAT, Visceral adipose tissue; VAT, Visceral adipose tissue; VAT, Visceral adipose tissue; SAT, Subcutaneous adipose tissue; VSR, Visceral-to-subcutaneous fat mass ratio; TAFA, Total abdominal fat mass.

### Development and validation of predictive models

**Figure 2:**
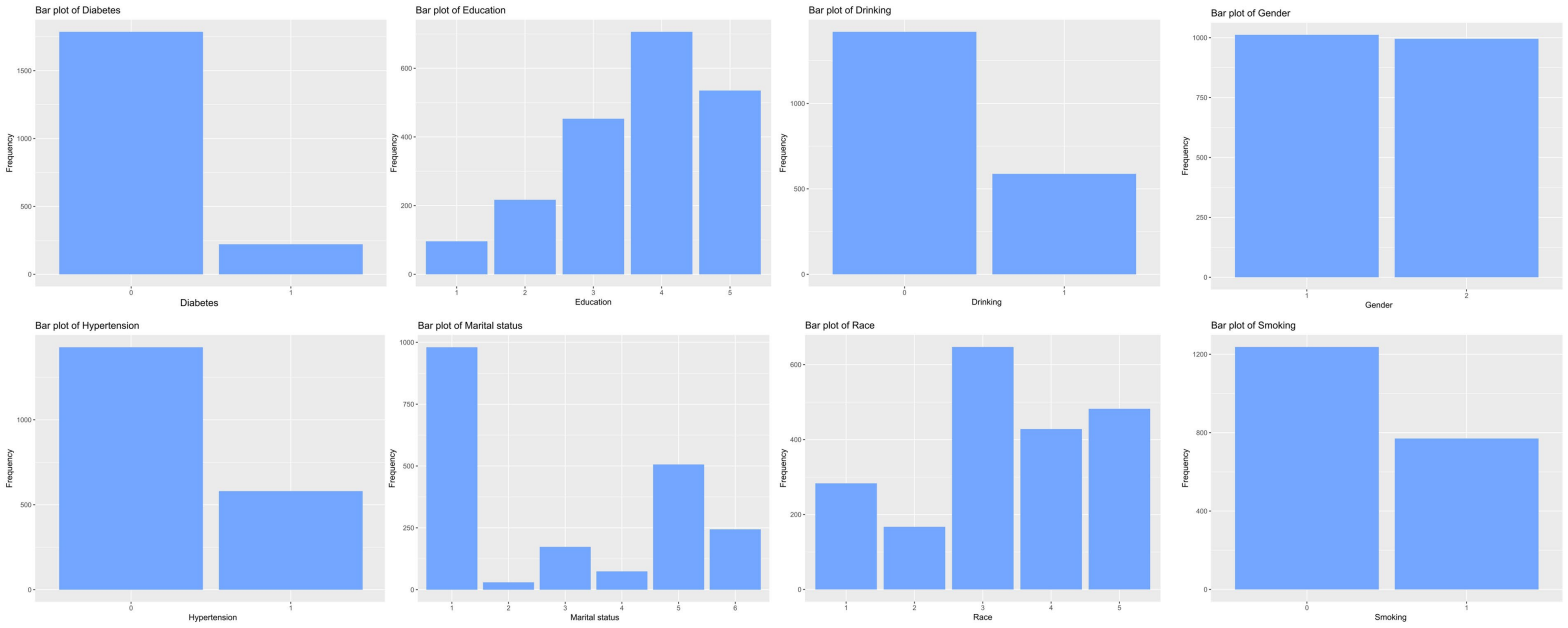
Characteristics of the distribution of categorical variables.

**Figure 3:**
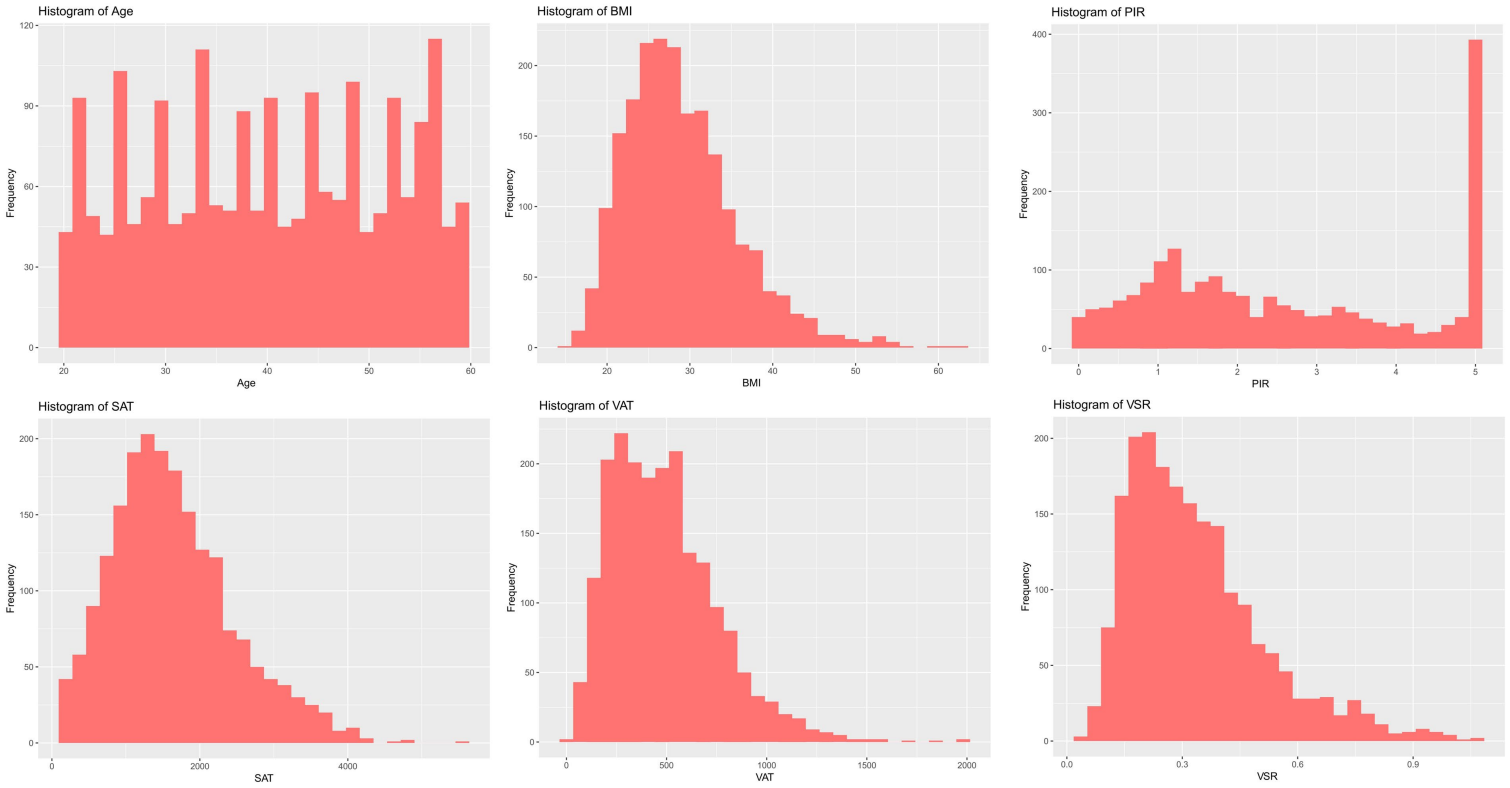
Characteristics of the distribution of continuous variables.

**Figure 4:**
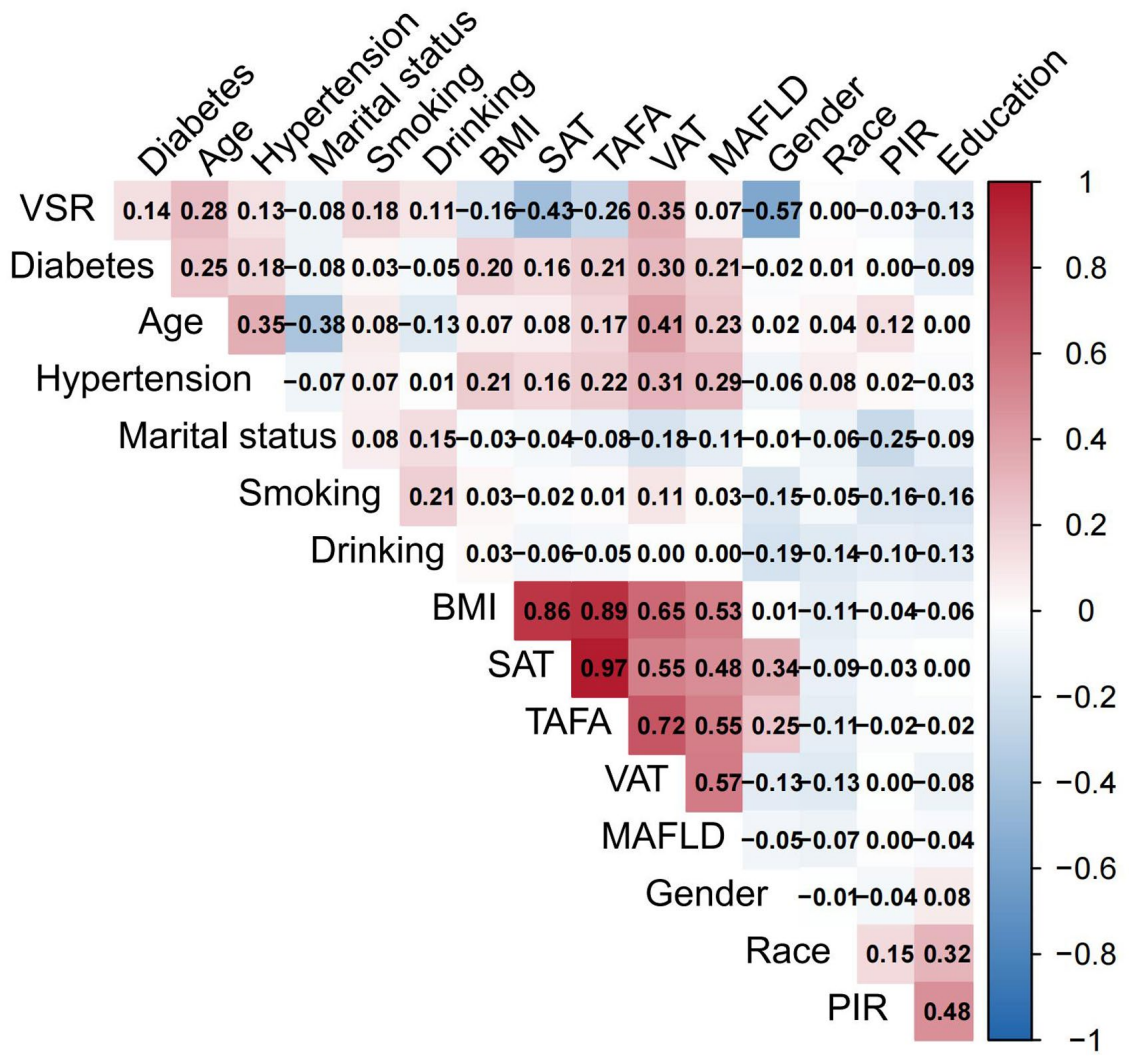
Evaluation of feature relevance.

**Figure 5:**
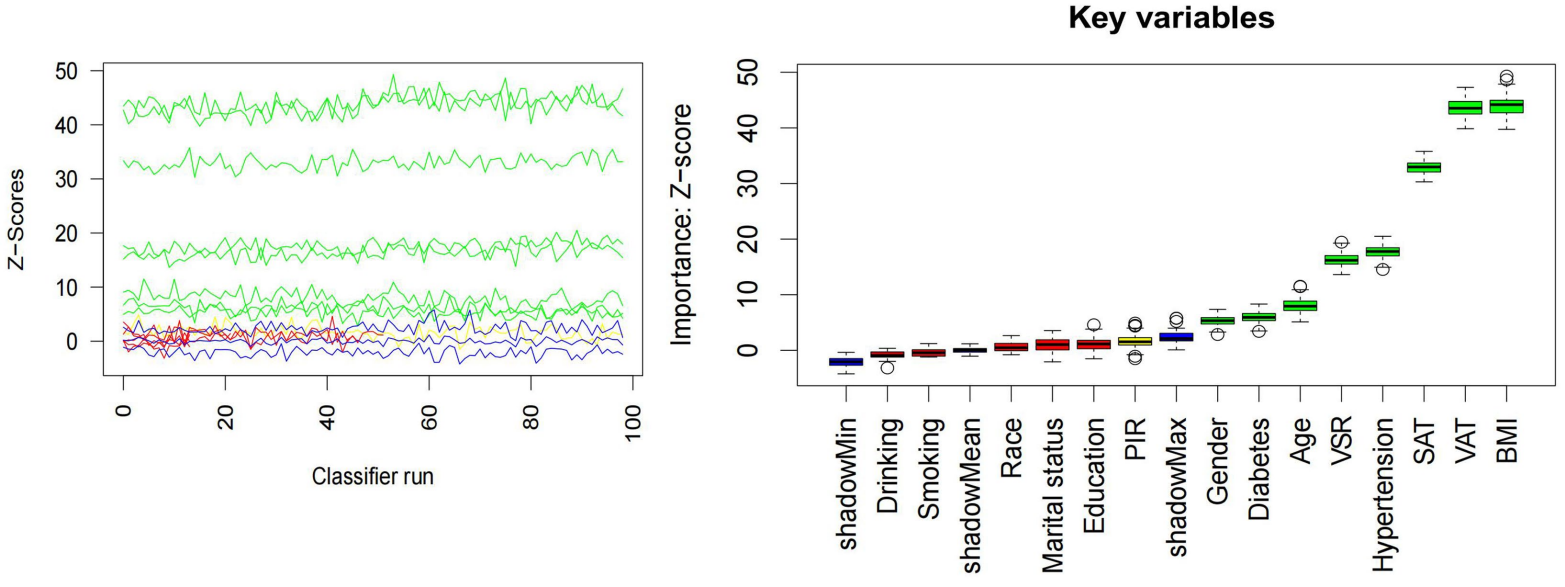
Boruta’s algorithm.

The distributions of all candidate variables were visualized (Figures 2, 3), and inter-variable correlations were examined using Pearson correlation coefficients (Figure 4). Multicollinearity was assessed by calculating the VIF; variables exhibiting high multicollinearity—TAFA—were excluded from further analysis. Subsequently, the remaining variables were subjected to feature selection using the Boruta algorithm. This method identified the top 10 features most strongly associated with MAFLD: VAT, BMI, SAT, VSR, hypertension, diabetes mellitus, age, gender, PIR, and marital status. These features were retained for subsequent ML model development (Figure 5).

**Figure 6:**
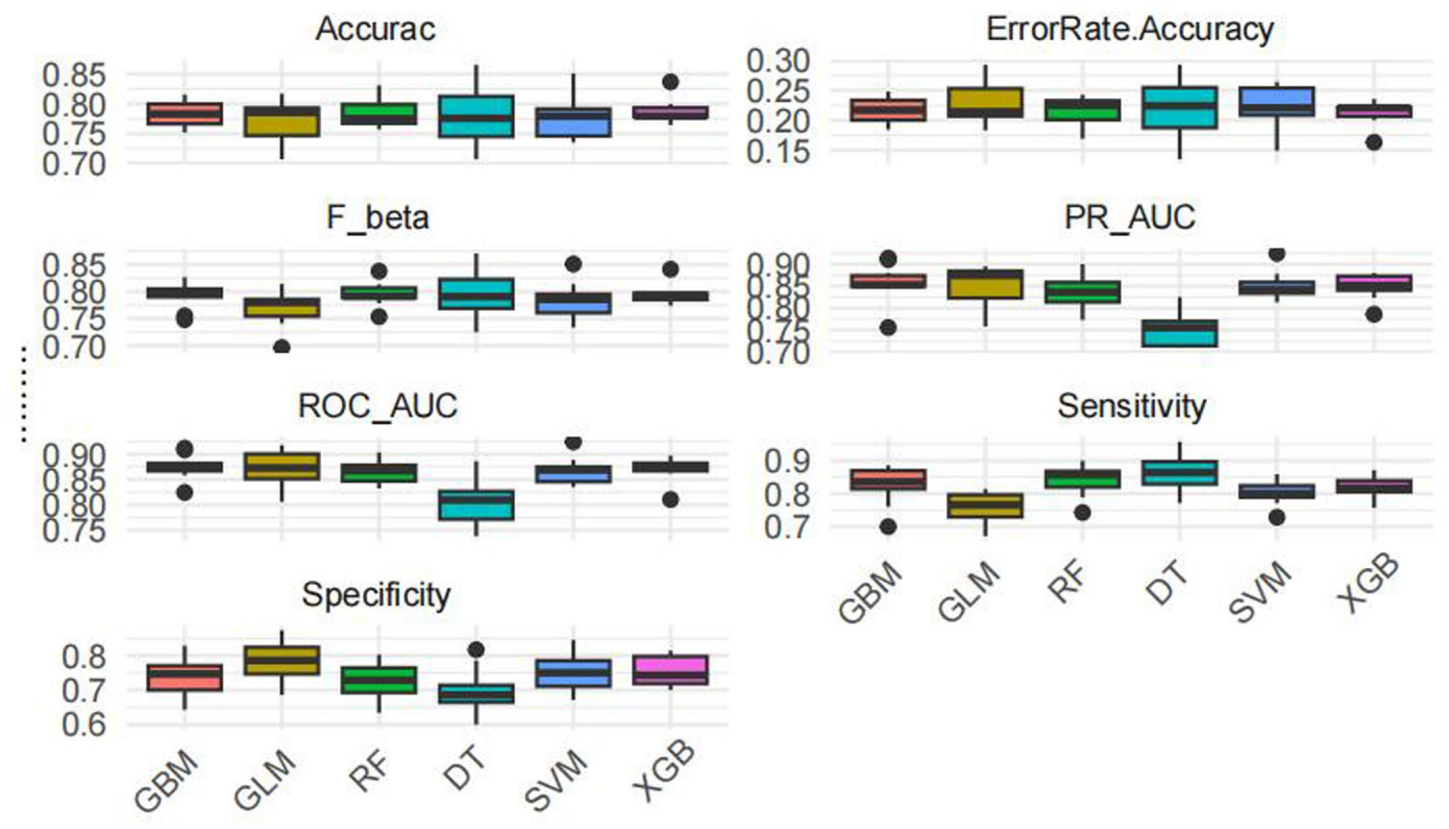
Ten-fold cross validation results.

**Figure 7:**
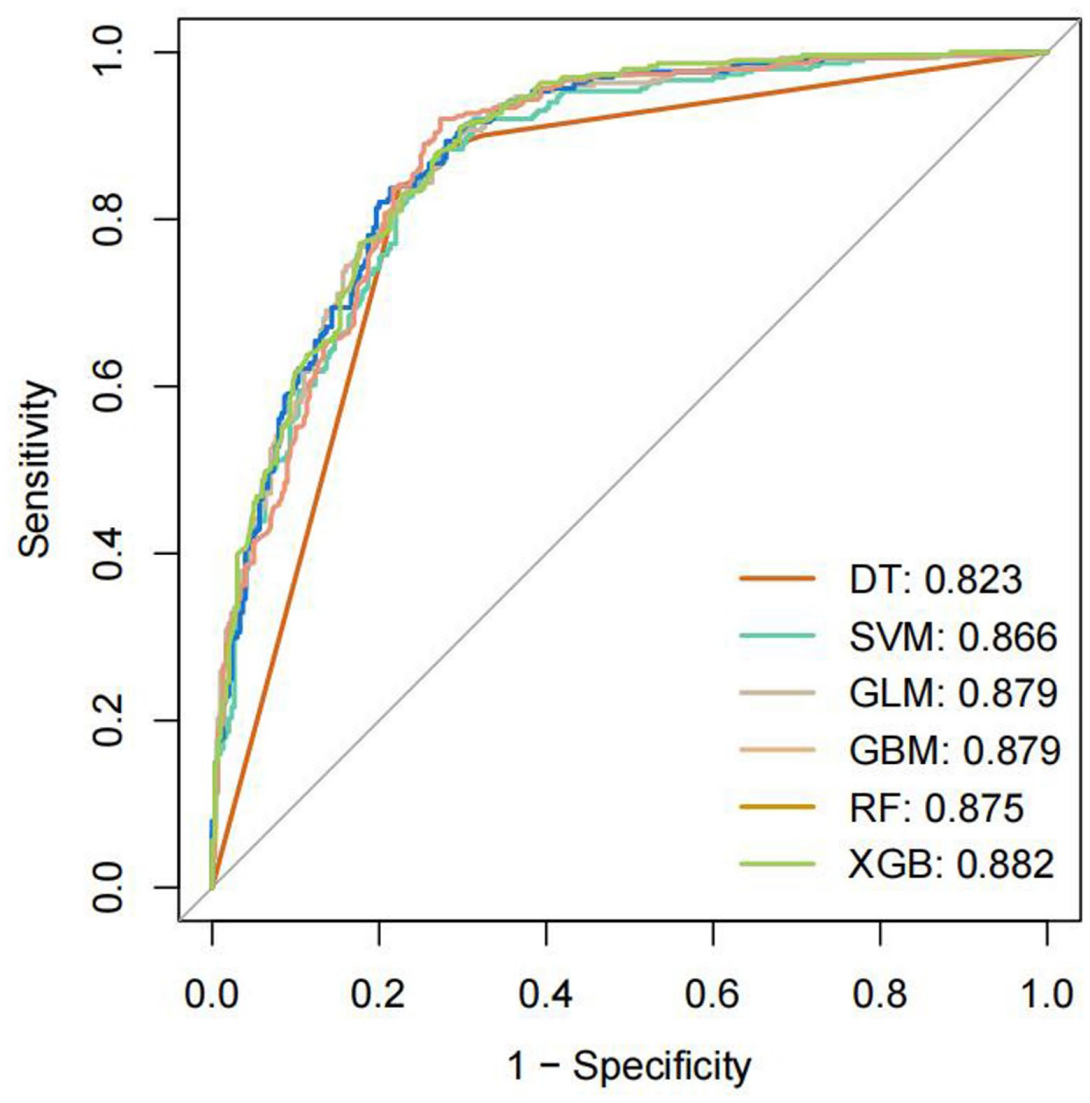
Receiver operating characteristic curve.

**Figure 8:**
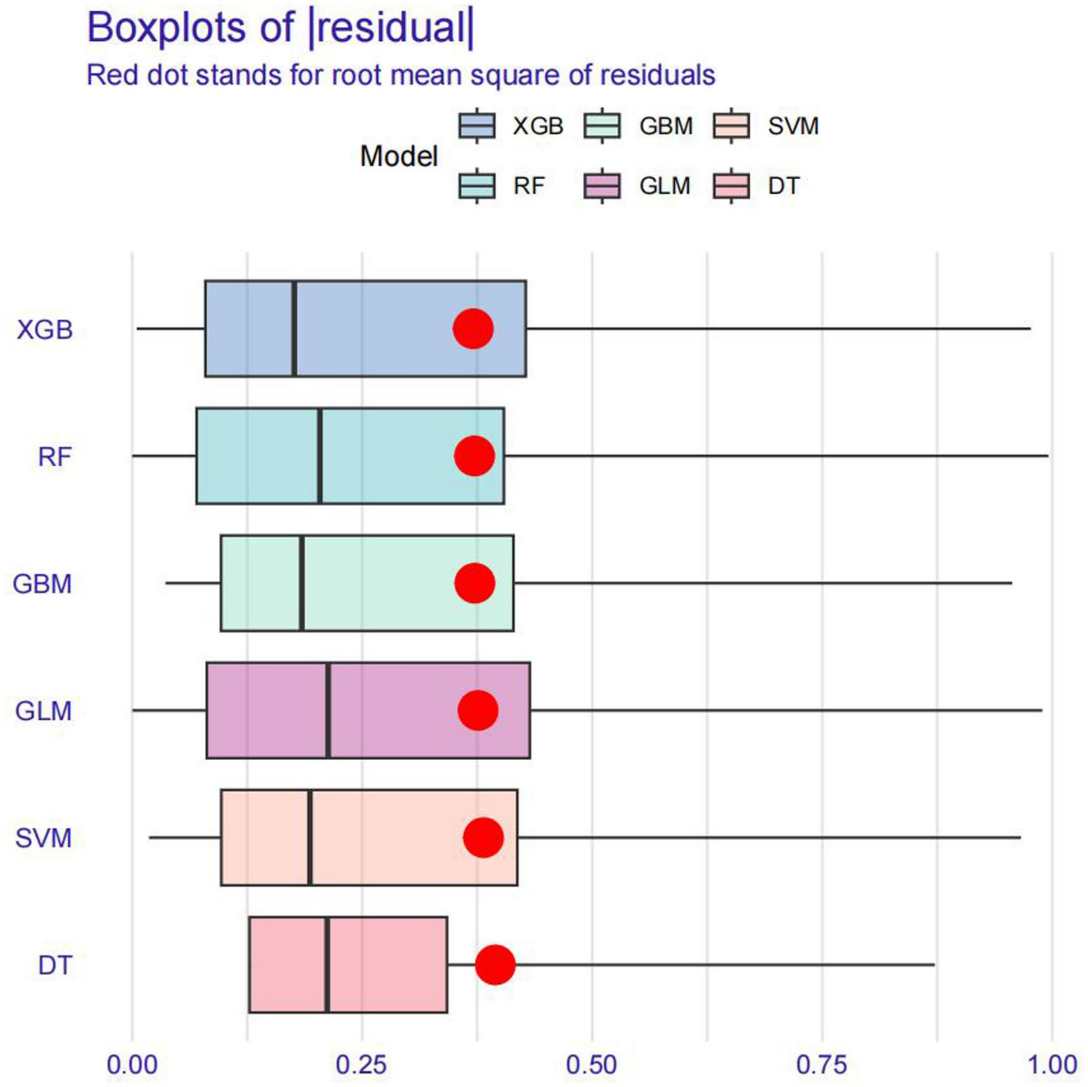
Residual analysis plot.

**Figure 9:**
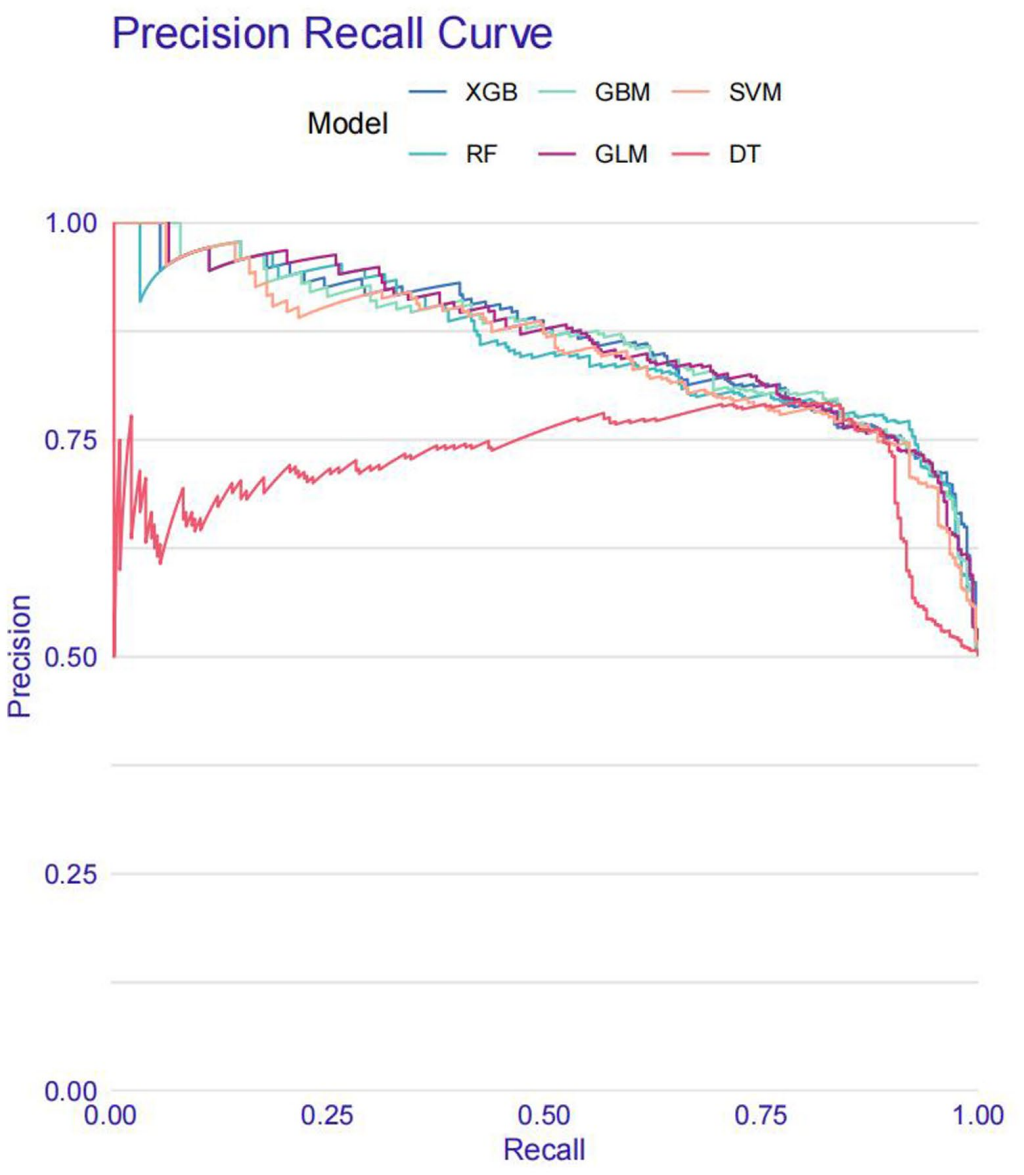
Recall curve.

Model training was conducted in two stages. First, 10-fold cross-validation was applied to the training set to evaluate internal model performance. Subsequently, the validation set was used to assess external performance and identify the optimal model through comparative analysis. Table 2 and Figure 6 summarized the prediction performance of the six ML models in the training set—DT, SVM, GLM, GBM, RF and XGBoost. Key evaluation metrics included the AUC, sensitivity, specificity, accuracy, F-beta score, and AUPRC, all reported as mean values. Among the six models, the GBM algorithm demonstrated the highest discriminative power in the training set, achieving the highest AUC (0.875), which was significantly superior to the other models (*p* = 0.005). It also achieved the best AUPRC (0.857, *p* < 0.001), while maintaining a favorable balance between sensitivity (0.826) and specificity (0.741). In terms of accuracy (0.784), GBM ranked jointly second with RF, following XGBoost. In the validation set (Table 3; Figure 7), XGBoost, GLM, and GBM exhibited comparable generalization performance. XGBoost achieved the highest AUC (0.882) and specificity (0.910), although its sensitivity was moderate (0.703), and it required a considerably lower optimal decision threshold (0.378) compared to GBM. Both GBM and GLM achieved AUC values of 0.879, tying for second place. GLM demonstrated the highest specificity (0.890) but the lowest sensitivity (0.717), while GBM maintained the most balanced performance with a sensitivity of 0.787 and specificity of 0.837. Residual analysis based on absolute error values (Figure 8) revealed that the GBM model exhibited a relatively narrow residual distribution, indicating greater stability and lower variance. Its median residual value was the lowest among all models, reflecting smaller average prediction errors and higher consistency. In contrast, the residuals of the XGBoost model showed greater variability, as indicated by a wider boxplot. We further evaluated the models using recall curves (Figure 9). We found that the recall curves of the GBM, XGB and RF models perform more smoothly. At high recall, GBM and XGB are able to maintain a high precision rate, while RF performs relatively stable but slightly inferior to the first two. Overall, the GBM model demonstrated superior robustness and stability. Its AUC remained consistent between the training (0.875) and validation (0.879) sets, and its sensitivity (0.826 vs. 0.787) and specificity (0.741 vs. 0.837) showed minimal fluctuation, indicating reliable and generalizable predictive performance.

**Table 2:** Six machine learning model metrics for predicting MAFLD in the training set.

Model	AUC	Specificity	Sensitivity	Accuracy	F beta	AUPRC
DT	0.803	0.697	0.859	0.778	0.795	0.750
SVM	0.867	0.752	0.804	0.778	0.784	0.850
GLM	0.871	0.785	0.758	0.772	0.769	0.850
GBM	0.875	0.741	0.826	0.784	0.792	0.857
RF	0.864	0.728	0.839	0.784	0.795	0.837
XGB	0.871	0.755	0.819	0.787	0.794	0.850
*p*-value	0.005^b^	0.003^a^	0.002^a^	0.903^a^	0.342^a^	<0.001^b^

DT, Decision Tree; SVM, Support Vector Machine; GLM, Generalized Linear Model; GBM, Gradient Boosting Machine; RF, Random Forest; XGB, XGBoost.

a ANOVA test.

b Kruskal-Wallis.

**Table 3:** Six machine learning model metrics for predicting MAFLD in the test set.

Model	AUC	Best threshold	Specificity	Sensitivity	Accuracy
DT	0.823	0.638	0.837	0.777	0.807
SVM	0.866	0.464	0.874	0.733	0.804
GLM	0.879	0.381	0.890	0.717	0.804
GBM	0.879	0.517	0.837	0.787	0.812
RF	0.875	0.413	0.920	0.727	0.824
XGB	0.882	0.378	0.910	0.703	0.807

DT, Decision Tree; SVM, Support Vector Machine; GLM, Generalized Linear Model; GBM, Gradient Boosting Machine; RF, Random Forest; XGB, XGBoost.

### SHAP interpretation of the optimal machine learning model

**Figure 10:**
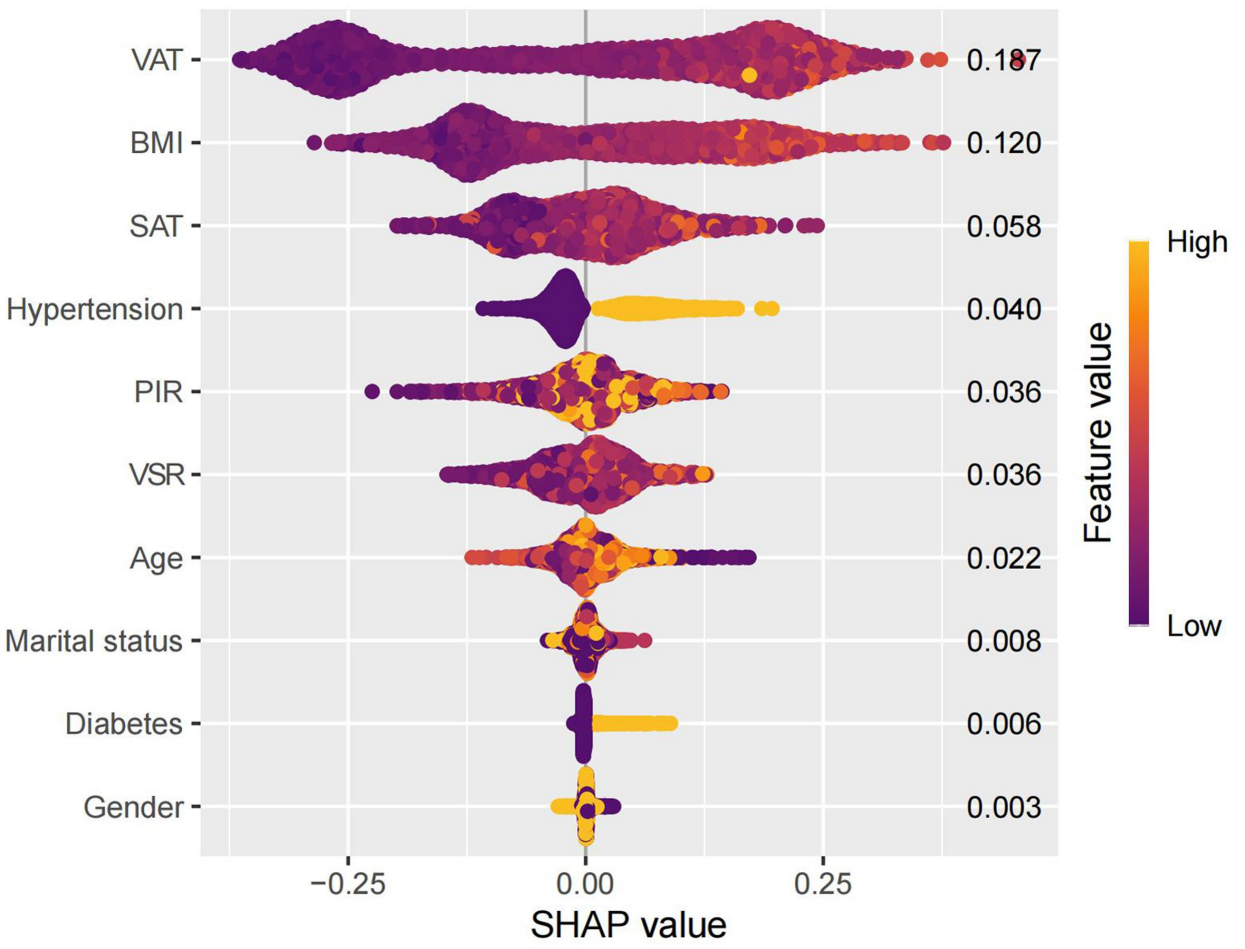
Swarm diagram.

**Figure 11:**
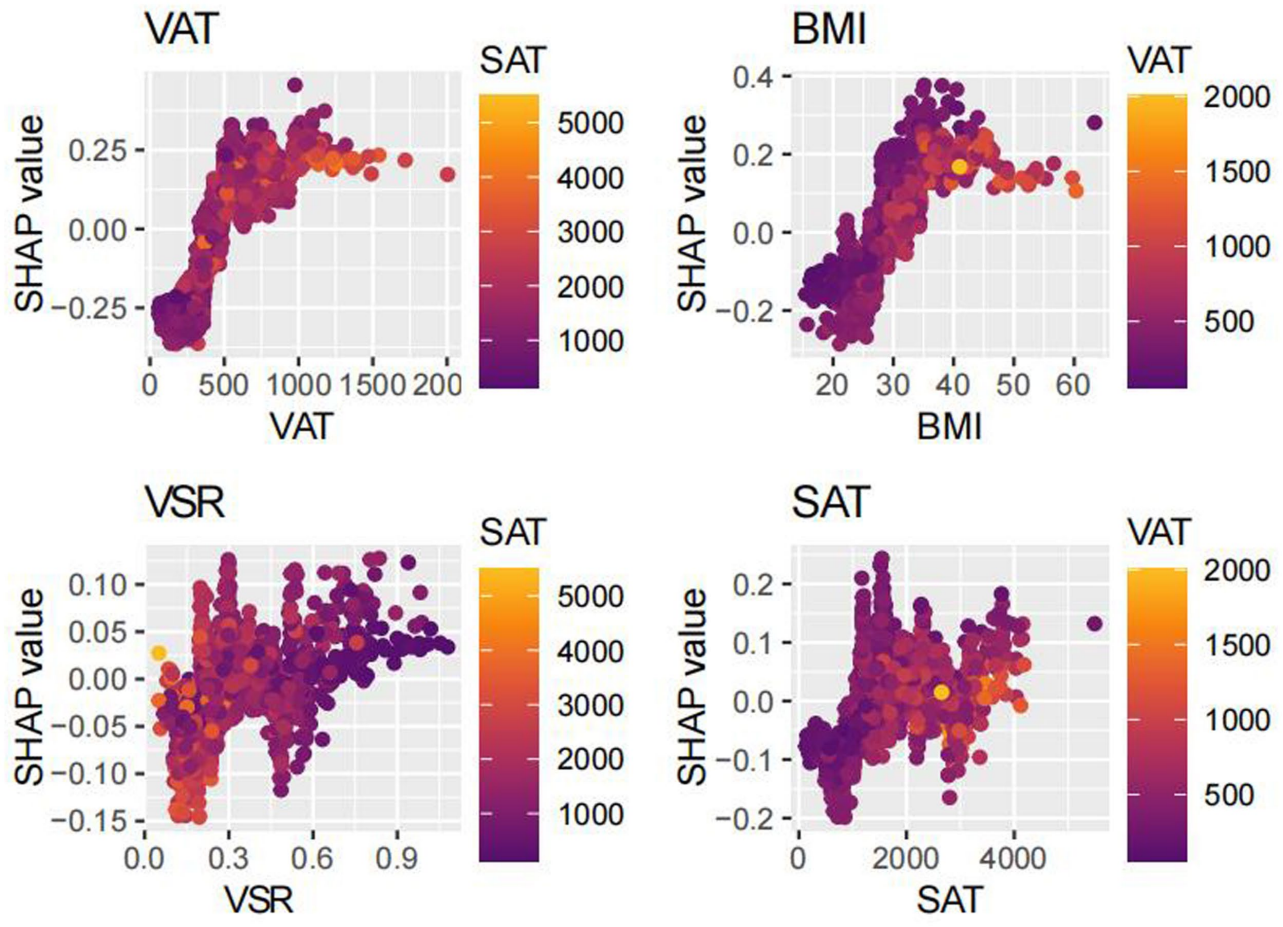
Dependency diagram.

SHAP analysis was employed to interpret the contributions of individual features to MAFLD prediction within the optimal machine learning model (Figure 10). The SHAP summary plot revealed that VAT, BMI, and SAT were the most influential predictors, with mean absolute SHAP values of 0.187, 0.120, and 0.058, respectively. Although the VSR demonstrated a lower SHAP value (0.036), it remained among the top 10 most important features. To further elucidate the relationships between individual features and model output, SHAP dependence plots were constructed (Figure 11). These plots showed that increasing VAT and BMI values were associated with rising SHAP values, indicating a higher predicted probability of MAFLD. Among them, VAT exerted the strongest marginal effect. Although SAT contributed less than VAT and BMI, it was still a meaningful predictor. In contrast, the impact of VSR on model predictions was relatively modest, suggesting a more limited role in classification performance.

## Discussion

In this study, we leveraged interpretable ML approaches to explore the association between body composition metrics and MAFLD using data from the 2017–2018 NHANES. Among the six ML algorithms evaluated, the GBM model demonstrated the most favorable overall performance. It achieved the highest AUC (0.879) in the validation set, closely mirroring its performance in the training set (AUC = 0.875). Additionally, the model exhibited minimal fluctuations in sensitivity and specificity across both datasets, underscoring its robustness, generalizability, and predictive reliability.

Using SHAP, we quantified the relative contributions of each selected feature to the model’s predictions. VAT, BMI, and SAT emerged as the most influential predictors, highlighting the central role of abdominal fat distribution in MAFLD pathogenesis. To the best of our knowledge, this is the first study to systematically assess the predictive value of detailed body composition metrics for MAFLD using machine learning techniques. The proposed model incorporates readily obtainable demographic, lifestyle, and clinical variables, enhancing both its predictive accuracy and its potential utility in routine clinical practice and population-level screening.

Our model identified VAT, BMI, and SAT as the most important predictors of MAFLD, aligning with existing evidence on the differential roles of adipose tissue depots in disease pathogenesis. Although BMI remains a widely used clinical measure of general obesity (19, 20), it fails to distinguish between lean mass and fat mass, and does not capture inter-individual differences in fat distribution (21). Emerging evidence suggests that the distribution of adipose tissue—particularly the accumulation of visceral fat—is more strongly associated with metabolic dysfunction and fatty liver disease than total fat mass alone (23–26). Notably, excess visceral adiposity has also been observed in individuals with normal BMI, a phenotype often referred to as “metabolically obese normal weight” or lean MAFLD. These individuals frequently exhibit greater insulin resistance and more advanced hepatic fibrosis (43), underscoring the critical role of VAT in disease progression, independent of overall body size.

Accordingly, the identification of VAT as the most important predictor in our model is biologically plausible. Visceral adipose tissue is metabolically active and, when excessively accumulated, increases the flux of FFAs into the portal circulation (44), thereby promoting hepatic lipid deposition and inducing insulin resistance (45, 46). Additionally, VAT secretes proinflammatory cytokines such as tumor necrosis factor-*α* (TNF-α), interleukin-6 (IL-6), and leptin, which activate hepatic Kupffer cells and hepatic stellate cells, contributing to hepatic inflammation and fibrogenesis (47–53). In contrast, SAT functions as a metabolic buffer or “lipid reservoir.” Under conditions of energy surplus, SAT preferentially expands through adipocyte hyperplasia to safely store excess lipids and mitigate ectopic fat deposition (44, 54). However, when the storage capacity of SAT is exceeded—due to genetic, epigenetic, or adipogenic constraints—surplus lipids may overflow into visceral compartments, including the liver (24, 30, 31, 36). Therefore, the identification of SAT as an important predictive feature underscores a critical pathophysiological concept: while total adiposity contributes to metabolic burden, it is the limited expandability of SAT and the consequent visceral fat accumulation that drives the development and progression of MAFLD. Collectively, these findings provide mechanistic validation for the high predictive value of obesity-related indices in our model and illustrate the advantage of machine learning approaches in capturing the complex, interdependent relationships among metabolic risk factors.

In this study, we implemented six classical ML algorithms—DT, SVM, GLM, GBM, RF, and XGBoost—to construct predictive models for MAFLD. This multi-model approach offers a comprehensive framework for risk stratification by capturing diverse patterns of feature–outcome relationships. Each algorithm is grounded in distinct theoretical principles and exhibits unique methodological advantages. DT constructs a hierarchical decision structure via recursive binary splits, effectively modeling nonlinear feature interactions. While highly interpretable, DTs are prone to overfitting and sensitive to data noise, necessitating pruning techniques to improve generalizability. GLM, commonly applied as logistic regression, assumes linear relationships between predictors and outcomes. It provides interpretable coefficients and serves as a robust baseline model, particularly under conditions of limited sample size or when feature effects are approximately linear. SVM identifies the optimal separating hyperplane with maximum margin between classes and can incorporate nonlinear kernels to capture complex decision boundaries. It is well-suited for high-dimensional and small-sample settings, though its model outputs are less intuitive than tree-based counterparts. RF and GBM represent ensemble learning strategies. RF leverages bagging to generate multiple decision trees trained on bootstrapped data subsets and aggregates their predictions via majority voting, thereby reducing variance and improving model stability. It also yields feature importance rankings, aiding interpretability. In contrast, GBM adopts a boosting strategy that sequentially minimizes prediction errors by fitting new models to the residuals of prior models. This enables the modeling of intricate nonlinear relationships but requires careful hyperparameter tuning—such as tree depth and learning rate—to avoid overfitting. XGBoost, an advanced and optimized version of GBM, integrates regularization and second-order gradient approximation to enhance training efficiency, reduce overfitting, and improve predictive accuracy. It has been widely adopted across biomedical classification tasks due to its robustness and computational scalability (55).

The selection of diverse machine learning algorithms for MAFLD prediction in this study is supported by both methodological rationale and prior empirical evidence. Given that MAFLD arises from a complex interplay of obesity, metabolic, and inflammation-related factors, which may exhibit nonlinear relationships and interaction effects, incorporating algorithms capable of capturing such complexities is essential. Traditional linear models may fail to identify intricate risk patterns that are better revealed by nonparametric or ensemble-based approaches. Previous studies have demonstrated the applicability and effectiveness of various ML models in fatty liver disease prediction. For instance, Qin developed decision tree, random forest, XGBoost, and support vector machine classifiers using physical examination and biochemical indicators to screen for NAFLD. Among these, the SVM model achieved the highest performance, with an AUC of approximately 0.85 and an accuracy of 80%, outperforming other models across multiple evaluation metrics (56). Similarly, Peng compared logistic regression, RF, GBM, XGBoost, and SVM models in predicting NAFLD and identified XGBoost as the top-performing algorithm, highlighting its clinical utility for early risk stratification (57). These findings support the validity of adopting multiple ML models in our framework. By leveraging the complementary strengths of different algorithms, our approach enhances discriminative performance while maintaining robustness and interpretability—crucial attributes for translation into real-world clinical or public health applications.

This study possesses several notable strengths. First, our predictive model demonstrated satisfactory discriminative performance in identifying individuals with MAFLD, indicating that body composition parameters can be effectively integrated into future clinical risk assessment frameworks. This finding supports the development of refined, obesity-based predictive strategies that extend beyond traditional anthropometric measures. Second, by incorporating multiple indices of body composition, our analysis underscores the critical role of fat distribution—rather than total adiposity alone—in the pathogenesis of MAFLD. This reinforces the clinical and public health imperative to shift the focus from general obesity metrics such as BMI toward a more nuanced evaluation of adipose tissue distribution. Such an approach may improve the accuracy of MAFLD screening and raise awareness of obesity-related phenotypic heterogeneity in disease development. Third, the GBM model exhibited strong translational potential. Its capacity for individualized risk estimation facilitates early identification of high-risk populations and the implementation of targeted preventive interventions aimed at mitigating progression to advanced liver disease. In resource-constrained healthcare environments, the model may assist in optimizing clinical resource allocation—for instance, by prioritizing high-risk individuals for advanced imaging modalities such as magnetic resonance imaging (MRI), thereby improving cost-effectiveness. Moreover, interpretability tools such as SHAP-derived feature importance can enhance clinician–patient communication by visualizing individual risk drivers. This may help increase patients’ understanding of their personal risk profiles and motivate adherence to lifestyle modifications, further bridging the gap between predictive analytics and actionable interventions in routine care.

Nevertheless, it is imperative to acknowledge the limitations inherent in the study. Firstly, histological evidence from liver biopsies is required for a definitive diagnosis of MAFLD. However, large population studies are difficult to perform invasively. Despite the utilization of FibroScan® transient elastography as an alternative in this study (a technique which has been demonstrated to possess clinical validity), the potential for the introduction of diagnostic bias remains a concern. Secondly, despite the Oral Glucose Tolerance Test (OGTT) representing a pivotal criterion for the diagnosis of diabetes, the absence of data pertaining to this indicator in the 2017–2018 NHANES cycle may have resulted in the under-recognition of cases of diabetes. Furthermore, as the data were derived from the NHANES cross-sectional survey in a single country, there are limitations in terms of sample representativeness and the applicability of the model to other populations. The model was trained and validated exclusively on an internal dataset, with no external validation performed using an independent cohort. This may be problematic due to potential differences in characteristics between populations of different races or regions, which could compromise the generalization ability of the model. Further assessment is required to ascertain the model’s generalization capability. It is important to note that the cross-sectional design of this study precluded the determination of whether the observed associations were causal or not. It is therefore essential that future longitudinal studies are conducted in order to more accurately assess the causal associations between obesity indicators and the development of MAFLD. The SHAP method is predicated on the assumption of independence of characteristics in interpreting the model. Despite the exclusion of highly correlated variables, residual correlations may still affect the interpretation of results.

## Conclusion

In this study, we developed predictive models for MAFLD using six machine learning algorithms: DT, SVM, GLM, GBM, RF, and XGBoost. Among these, the GBM model demonstrated the most favorable overall performance, achieving high discriminative accuracy and stability across both training and validation datasets. Furthermore, SHAP analysis provided interpretable insights into feature contributions, with VAT emerging as the most important predictor of MAFLD risk. These findings underscore the utility of integrating advanced machine learning techniques with detailed body composition metrics to improve early risk stratification and guide targeted interventions in clinical and public health contexts.

## Data Availability

The datasets presented in this study can be found in online repositories. The names of the repository/repositories and accession number(s) can be found at: https://wwwn.cdc.gov/nchs/nhanes/default.aspx.
